# Catechins Variously Affect Activities of Conjugation Enzymes in Proliferating and Differentiated Caco-2 Cells

**DOI:** 10.3390/molecules21091186

**Published:** 2016-09-07

**Authors:** Kateřina Lněničková, Eliška Procházková, Lenka Skálová, Petra Matoušková, Hana Bártíková, Pavel Souček, Barbora Szotáková

**Affiliations:** 1 Department of Biochemical Sciences, Faculty of Pharmacy, Charles University, Hradec Králové CZ-50005, Czech Republic; lnenickk@faf.cuni.cz (K.L.); prochae1@faf.cuni.cz (E.P.); skaloval@faf.cuni.cz (L.S.); matousp7@faf.cuni.cz (P.M.); barth3aa@faf.cuni.cz (H.B.); 2 Toxicogenomics Unit, Centre of Toxicology and Health Safety, National Institute of Public Health, Prague CZ-10042, Czech Republic; pavel.soucek@szu.cz

**Keywords:** green tea extract, epigallocatechin gallate, sulfotransferase, glutathione S-transferase, catechol-*O*-methyltransferase, UDP-glucuronosyl transferase, enterocyte-like cells, cancer cells

## Abstract

The knowledge of processes in intestinal cells is essential, as most xenobiotics come into contact with the small intestine first. Caco-2 cells are human colorectal adenocarcinoma that once differentiated, exhibit enterocyte-like characteristics. Our study compares activities and expressions of important conjugation enzymes and their modulation by green tea extract (GTE) and epigallocatechin gallate (EGCG) using both proliferating (P) and differentiated (D) caco-2 cells. The mRNA levels of the main conjugation enzymes were significantly elevated after the differentiation of Caco-2 cells. However, no increase in conjugation enzymes’ activities in differentiated cells was detected in comparison to proliferating ones. GTE/EGCG treatment did not affect the mRNA levels of any of the conjugation enzymes tested in either type of cells. Concerning conjugation enzymes activities, GTE/EGCG treatment elevated glutathione S-transferase (GST) activity by approx. 30% and inhibited catechol-*O*-methyltransferase (COMT) activity by approx. 20% in differentiated cells. On the other hand, GTE as well as EGCG treatment did not significantly affect the activities of conjugation enzymes in proliferating cells. Administration of GTE/EGCG mediated only mild changes of GST and COMT activities in enterocyte-like cells, indicating a low risk of GTE/EGCG interactions with concomitantly administered drugs. However, a considerable chemo-protective effect of GTE via the pronounced induction of detoxifying enzymes cannot be expected as well.

## 1. Introduction

The Caco-2 cell line, derived from human epithelial colorectal adenocarcinoma, has been extensively used for in vitro models. After reaching confluence, the cells are able to differentiate into enterocyte-like cells, form tight junctions, and express many transporters normally found in the small intestine [1], thus Caco-2 cells are usually used as a tool to investigate intestinal transport. In addition, the Caco-2 cell line can serve as a model for the study of the metabolism of xenobiotics and effects in the intestine. With respect to the fact that most drugs, food supplements, and other xenobiotics are administered orally and absorbed in the small intestine, the determination of the metabolism and action of xenobiotics in intestinal cells is very important. In addition, the Caco-2 cell line allows the study and comparison of the effects of xenobiotics both in proliferating and differentiated cells. Several articles have mapped differences regarding the expression of enzymes, including xenobiotic metabolizing enzymes, between proliferating and differentiated Caco-2 cells [2,3,4,5]. Generally, during cellular differentiation an increased expression of enzymes involved in the metabolism of xenobiotics has been detected. Nevertheless, information about the activities of xenobiotic metabolizing enzymes, especially conjugation enzymes, during the differentiation process is limited.

Conjugation enzymes, which catalyze phase II of xenobiotics metabolism, bind endogenous compounds to xenobiotics or their metabolites, which arise in phase I via oxidation, reduction, or hydrolysis. Conjugation usually introduces hydrophilic ionizable functional groups (e.g., glutathione, glucuronic acid, sulfate) to the molecule of a xenobiotic, thus making it more polar and less active. Conjugation enzymes are therefore extremely important in the defense of organisms against potentially harmful xenobiotics [6,7]. For that reason, modulation (inhibition, induction) of the activity of these enzymes can have a pronounced pharmacological/toxicological impact. Many natural compounds (e.g., flavonoids) could modulate the expression and/or activity of conjugation enzymes [8,9]. In spite of this fact, the modulatory effects of flavonoids on conjugation enzymes, with the exception of glutathione S-transferases, have not as of yet been sufficiently studied.

Flavonoids are natural polyphenolic compounds found ubiquitously in fruits, vegetables, and many plant products. Green tea, one of the most consumed beverages in the world, contains many flavonoids, in particular catechins ((−)-epigallocatechin gallate (EGCG), (−)-epigallocatechin, (−)-epicatechin gallate, and (−)-epicatechin)); these flavonoids have been studied intensively for various beneficial effects to human health [10]. EGCG, the most abundant catechin (50%–60%) in green tea, has so far been found to be responsible for most green tea health-promoting ability [11,12]. The popularity of green tea has led to the production of various food supplements with concentrated green tea extracts and the consumption thereof in excessive amounts. Especially in high concentration, however, catechins may modulate the activity of xenobiotic metabolizing enzymes in both a desired and undesired manner, bringing significant pharmacological and/or toxicological consequences [13]. As only a small part of the catechins that are consumed are absorbed, the intestine is exposed to the highest catechins concentrations, thus intestinal enzymes could be the most affected. In addition, the modulatory effect of catechins on xenobiotic metabolizing enzymes may differ in proliferating and differentiated intestinal cells. Knowledge of these differences could prove significant in the evaluation of risks and/or benefits of the consumption of green tea catechins in view of their effect in proliferating and differentiated cells.

For these reasons the present in vitro study was designed to compare the activities and mRNA expression in Caco-2 proliferating (P) and differentiated (D) cells of the main conjugation enzymes glutathione S-transferases (GST), UDP-glucuronosyl transferases (UGT), sulfotransferases (SULT), and catechol-*O*-methyltransferases (COMT). To this end, the effects of green tea extract (GTE) and EGCG on cell viability and conjugation enzymes activities and mRNA levels in both types of cells were tested and compared.

## 2. Results

### 2.1. Activities and Expression of Conjugation Enzymes in P and D Caco-2 Cells

The specific activities of conjugation enzymes were assayed in subcellular fractions obtained from P and D cells homogenates, with no activity of UGT detected in either type of cells. The activities of other enzymes (GST, COMT, SULT) were detectable. No significant difference in GST and COMT specific activity was observed between P and D cells; the only significant difference found was in SULT activity. The specific activity of SULT in the D cells was significantly (*p* < 0.05) decreased by 28.5% compared with P cells (see Table 1).

The quantitative real-time PCR method was used to compare mRNA expression of selected conjugation enzymes in P and D Caco-2 cells. For this purpose, primers specific for SULT1A1/3, COMT, GSTP1, GSTA1/2, UGT1A, and glyceraldehyde-3-phosphate dehydrogenase (GAPDH serving as a reference gene) were used. The quantification of individual conjugation enzymes mRNA in Caco-2 cells showed that the D cells in comparison to P cells showed a significantly enhanced mRNA level of SULT1A1/3, COMT, GSTP1, GSTA1/2, and UGT1A in (see Table 1). The highest differentiation-mediated increase in mRNA levels was observed in the case of GSTA1/2 (9.15-times).

### 2.2. The Effect of GTE and EGCG on Cell Viability/Proliferation

**Figure 1:**
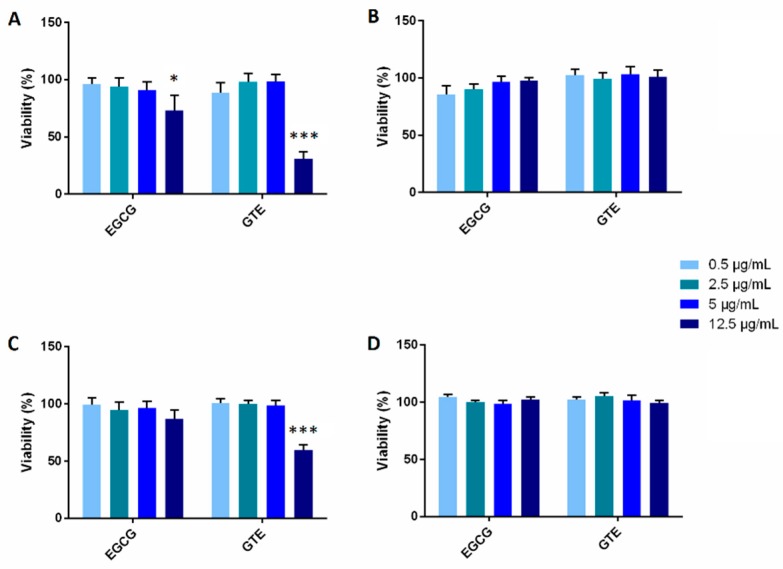
Viability of cells after exposure to epigallocatechin gallate (EGCG) and green tea extract (GTE) in four concentrations. MTT assay (**A**,**B**) and neutral red uptake (NRU) test (**C**,**D**) of proliferating (**A**,**C**) and differentiated (**B**,**D**) Caco-2 cells. The viabilities of treated cells are expressed as the percentage of untreated controls (100%). Values represent means ± SD of three samples. Significance of differences from the control was determined by ANOVA followed by a Dunnett’s test (* *p* < 0.05, *** *p* < 0.001).

The P and D cells were exposed to GTE and to EGCG in the concentration range 0–12.5 µg/mL. After 72-h treatment, cell viability was determined using an MTT assay and neutral red uptake (NRU) test (Figure 1). In the D cells, neither GTE nor EGCG had an effect, while a significant antiproliferative effect of GTE (at the highest concentration) was observed in the P cells. Based on the obtained results, two non-toxic concentrations (1 µg/mL and 5 µg/mL) were chosen for further experiments.

### 2.3. The Effect of GTE and EGCG on Conjugation Enzymes Activities and Expressions in P and D Caco-2 Cells

GST, SULT, and COMT specific activities were measured in cytosol from the control and GTE or EGCG influenced P and D cells. No UGT activity was detected after GTE/EGCG treatment.

**Figure 2:**
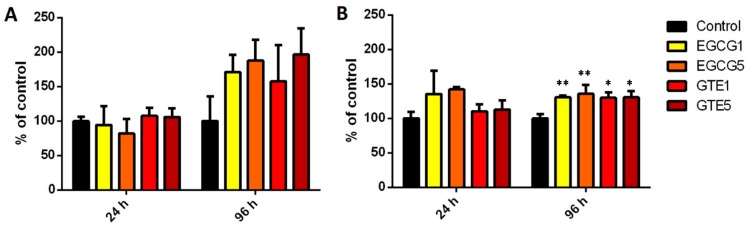
GST activity in (**A**) proliferating and (**B**) differentiated Caco-2 cells incubated with 1 and 5 µg/mL EGCG and GTE for 24 and 96 h. The bars represent means ± SD after at least three independent measurements. Significance of differences from the control was determined by ANOVA followed by a Dunnett’s test (* *p* < 0.05, ** *p* < 0.01).

As shown in Figure 2, after 24-h treatment neither GTE nor EGCG influenced GST activity in the P and D cells. After 96-h treatment, GST activity was increased. In the P cells this elevation was insignificant due to high variability. A significant increase in GST activity was observed in the D cells after 96-h treatment with either GTE or EGCG treatments (EGCG1 130% ± 2%; EGCG5 135% ± 11% of control; GTE1 120% ± 14%; GTE5 131% ± 6% of control).

**Figure 3:**
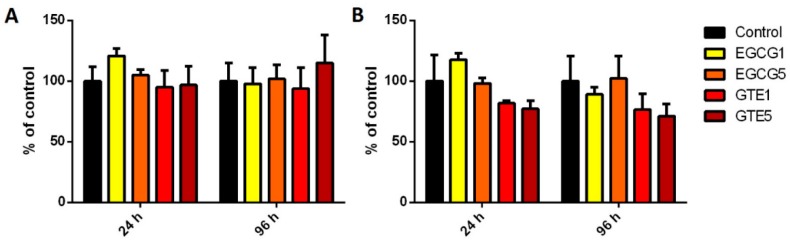
SULT activity in (**A**) proliferating and (**B**) differentiated Caco-2 cells incubated with 1 and 5 µg/mL EGCG and GTE for 24 and 96 h. The bars represent means ± SD of at least three independent measurements. Significance of differences from the control was determined by ANOVA followed by a Dunnett’s test.

Concerning cytosolic SULT activity (see Figure 3), no significant effect of GTE or EGCG was observed in P and D cells. However, GTE in D cells caused slight (insignificant) decreases of SULT activity in both concentrations and both incubation times. On the other hand, 24-h treatment with EGCG in lower concentration (1 µg/mL) led to mild (insignificant) increases in the SULT specific activity in both P and D cells.

**Figure 4:**
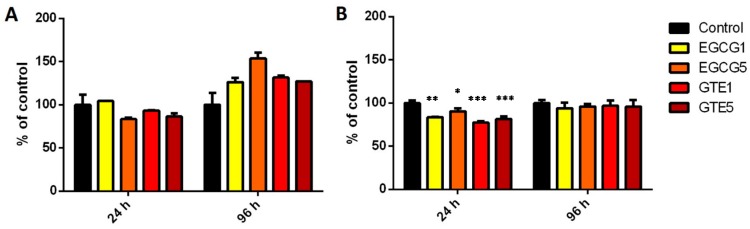
COMT activity in (**A**) proliferating and (**B**) differentiated Caco-2 cells incubated with 1 and 5 µg/mL EGCG and GTE for 24 and 96 h. The bars represent means ± SD of at least three independent measurements. Significance of differences from the control was determined by ANOVA followed by a Dunnett’s test (* *p* < 0.05, ** *p* < 0.01, *** *p* < 0.001).

The effect of GTE and EGCG on COMT activity in the P and D cells is presented in Figure 4. The 24-h treatment with EGCG as well as GTE caused a significant decrease in COMT activity in D cells (EGCG1 81% ± 3%, EGCG5 88% ± 0.6%; GTE1 75% ± 1%, GTE5 79% ± 0.4% of control) but not in P cells. 96-h Treatment with GTE and EGCG did not affect COMT activity in both cell types.

With the aim of determining the effect of GTE/EGCG on the expression of the conjugation enzymes, the P and D cells were exposed to higher concentrations (5 µg/mL) of EGCG and GTE for 12, 24, and 96 h. The qPCR results did not show significant changes in selected genes’ expression after GTE and EGCG treatment in either type of cells (data not shown).

## 3. Discussion

The first aim of our study was to compare the activities and mRNA levels of the main conjugation enzymes in proliferating (P) and differentiated (D) Caco-2 cells, as information about xenobiotic metabolizing enzymes, especially conjugation enzymes, during the differentiation process is limited. Cells were considered differentiated at the 18th day after they reached confluence [2]. Several previous studies have described the proteomic changes as well as the gene expression profiles of proliferating and differentiated Caco-2 cells [2,3,4,5]. In the mentioned studies, the upregulation of xenobiotic metabolizing enzymes expression during cellular differentiation was regularly detected. Buhrke et al. [2] reported GST induction at the mRNA and protein level. The qPCR method performed in our study showed similar results. GSTA1/2 and GSTP1 mRNA levels were significantly higher in D cells. When GST activity was measured, however, the P and D cells were seen to maintain similar GST activity. Regarding COMT expression during the differentiation of Caco-2 cells, no information has of yet been made available. In our study, COMT the mRNA level in the D cells was three-times higher, with the specific activity of COMT slightly but insignificantly augmented in D cells compared to P ones. No activity of UGTs was detected in either type of cells, but UGT1A mRNA was found at a level two-times higher in the D cells than in P cells. The mRNA expression of SULT1A1/3 has been found to be higher in D Caco-2 cells than in P ones, as previously published [2,14]. On the other hand, SULT activity was lower in D than in P cells. These discrepancies between mRNA levels and the activity of the corresponding enzyme could indicate that translation of some conjugation enzymes (especially of SULT1A1/3) might be substantially inhibited in D cells, but this hypothesis will need further verification.

Secondly, in the present work the effect of GTE and EGCG on cell viability and/or proliferation was tested and compared in P and D cells. The commercially available Polyphenon 60 (GTE), a defined purified form of green tea extract, and EGCG, the most abundant component of GTE, were used for this purpose. To find out the effect of catechins on P cell viability, GTE and EGCG were added to the incubation medium on the day the cells reached confluence and were cultivated for a further 72 h. To determine the influence of catechins on the D cells, we maintained the cells for 18 days after they reached confluence, at which time GTE and EGCG were added to the incubation medium for a further 72 h. Viability tests showed that catechins in the highest tested concentration (12.5 µg/mL) decreased the number of viable P cells, while the viability of D cells was not affected. These results indicate an antiproliferative effect of green tea in colon cancer cells, an effect of green tea catechins which has been reported in several cancer cell lines (e.g., human colorectal cancer cell lines (HCT-116 and SW-480), a human breast carcinoma cell line (MDA-MB-231), and human lung adenocarcinoma cell lines (NCI-H661, NCI-H441, and NCIH1299)) [15,16,17].

Another substantial part of our project was focused on the potential modulatory effect of GTE and EGCG on GST, SULT, COMT, and UGT in Caco-2 cells. All dietary polyphenols, including catechins, are substrates for biotransformation enzymes [18,19] and they may compete with other substrates (i.e., drugs, other xenobiotics, and endogenous substances) for the enzyme active site. In the human organism, catechins, including EGCG, undergo *O*-methylation, glucuronidation, sulfation, and ring-fission metabolism [13,18,20,21]. For that reason, the inhibition of COMT, UGT, and SULT via catechins is highly probable. In addition, catechins may also inhibit xenobiotic metabolizing enzymes in a non-competitive manner [12,22]. Moreover, catechins are also able to increase the expression of various xenobiotic metabolizing enzymes via several signal transduction pathways. Therefore, a high intake of catechins may lead to modulation of these enzymes [13]. Due to the very low bioavailability of consumed catechins, their highest concentration is presented in the small intestine [23]. Thus the highest probability of enzyme modulation could be predicted in intestinal cells.

One of beneficial impacts of green tea is represented by the up-regulation of GST, a process which is responsible for the elimination of potential toxic electrophiles [24]. This effect has been studied in many different model systems with various results [25,26,27,28]. In the present study, the modulatory effect of GTE and EGCG was tested and compared in P and D cells. Our findings, namely the elevation of GST activity after 96 h of incubation with catechins, are in accordance with most of these models. This effect was not confirmed at mRNA levels, with the content of GSTA1/2 and GSTP1 mRNA remaining unchanged.

The effect of catechins on SULT activities was found to be mild and insignificant. In the D cells, GTE slightly decreased SULT activities, while no inhibition was observed in the P cells. Previously, the inhibition of SULT activity by green tea beverages has been observed in studies with recombinant enzymes as well as in intestinal cell lines [29,30].

Concerning COMT, it was found that GTE and EGCG significantly decreased COMT activity only in the D cells after 24 h. On the other hand, mRNA levels showed no modulation. COMT activity was restored after longer exposure of D cells to GTE/EGCG, which may suggest biotransformation of GTE/EGCG substrates. A further decrease of COMT activity, possibly by competitive inhibition, might no longer occur. However, such an explanation could be only verified by metabolism studies. Lorenz et al. (2014) reported that a high dose of EGCG did not inhibit the COMT activity in red blood cells [31]. In other tissues (liver and intestine) and in different species (human, pig, rat, and mice) COMT inhibition by catechins has been described [21,32]. COMT is considered to be an important enzyme for the protection of cells from toxicity caused by catechols by the prevention of their conversion to quinones [33]. Therefore, the inhibition of COMT activity by catechins in enterocyte-like cells cannot be regarded as a desired effect of green tea catechins.

No UGT activity was detected in either type of cells after EGCG/GTE treatment, nor was the UGT1A mRNA level changed by EGCG/GTE action.

## 4. Materials and Methods

### 4.1. Chemicals and Reagents

Eagle’s minimum essential medium (EMEM), MEM non-essential amino acid solution, l-glutamine solution, l-(−)-norepinephrine, *S*-(5′-Adenosyl)-l-methionine (SAM), l-glutathione (GSH), 1-chloro-2,4-dinitrobenzene (CDNB), 3′-phosphoadenosine 5′-phosphate (PAP), 2-naphthol, menadione, and Polyphenon 60 (GTE), the defined form of GTE, were obtained from Sigma-Aldrich (Prague, Czech Republic). Fetal bovine serum and streptomycin sulfate were purchased from Invitrogen (Carlsbad, CA, USA). (−)-EGCG was supplied by Extrasynthese (Lyon, France). We bought ProtoScript^®^ II Reverse Transcriptase from NEB (Ipswich, UK), TriReagent from Biotech (Prague, Czech Republic), and the qPCR Core kit for SYBR Green I from Eurogentec (Seraing, Belgium). All other chemicals, HPLC or analytical grade, were obtained from Sigma-Aldrich.

### 4.2. Cultivation of the Caco-2 Cell Line

The human epithelial colorectal adenocarcinoma Caco-2 cell line (ATCC, supplier for Czech Republic: LGC Standards, Poland) was cultured in EMEM supplemented with 10% (*v*/*v*) heat-inactivated fetal bovine serum, 1% (*v*/*v*) non-essential amino acids, 1% (*v*/*v*) glutamine, and 0.5% penicillin/streptomycin. The cells were grown in Petri dishes or in 96-well plates for 3 (proliferating cells, P) and 21 (differentiated cells, D) days in a humidified atmosphere containing 5% CO_2_ at 37 °C. The medium was changed twice a week.

### 4.3. Tests of Cell Viability

The EGCG and GTE were pre-dissolved in DMSO. The concentration of DMSO in the medium was 0.1%. The cells were treated with various concentrations (0.5, 2.5, 5.0, 12.5 µg/mL) of EGCG or GTE in a culture medium for 72 h. Cells cultured in a medium with 0.1% DMSO were used as control samples. The viability of cells cultured in 96-well plates was assayed using the neutral red uptake (NRU) test or a MTT assay.

#### 4.3.1. NRU Test

After 72 h treatment, the medium was removed and 100 µL of neutral red-containing medium (40 µg/mL) was added into each well and the plates were incubated at 37 °C for an additional 3 h; then the cells were washed with phosphate buffered saline (PBS). The cells were fixed in a solution of 0.5% formaldehyde with 1% calcium chloride for 15 min. The neutral red dye was extracted from the viable cells with a solvent (50% ethanol with 1% acetic acid) by shaking for 30 min at room temperature. The absorbance of solubilized dye was measured using a spectrophotometer (Tecan Infinite M200, Tecan Group, Männedorf, Switzerland) at 540 nm. The viabilities of treated cells were expressed as the percentage of untreated controls (100%).

#### 4.3.2. MTT Assay

The mitochondria of living cells are able to reduce yellow MTT (3-(4,5-dimethylthiazol-2-yl)-2,5-diphenyltetrazolium bromide) to purple formazan. After 72 h exposure, 50 µL of MTT solution (3 mg of MTT in 1 mL of PBS) was added to each well. The plates were incubated at 37 °C for an additional 2 h, then the medium was removed and the formed formazan was dissolved in 50 µL of 0.08 M HCl in isopropanol by shaking for 30 min. The absorbance in each well was read at 595 nm using a spectrophotometer (Tecan Infinite M200).

### 4.4. Treatment of Caco-2 Cells with GTE and EGCG

These cells were grown in Petri dishes for 3 (P) or 21 (D) days, after which the cells were influenced by GTE and EGCG with a final concentration of 1 µg/mL (GTE1, EGCG1) or 5 µg/mL (GTE5, EGCG5) for 24 and 96 h. For mRNA quantification, the P and D cells were treated with 5 µg/mL of GTE and EGCG for 12, 24, and 96 h. The cells were treated with a single dose and the medium was not changed during treatment. At the end of incubation, the cells were washed with PBS and they were harvested into a 0.1 M sodium phosphate buffer (pH 7.4) or TriReagent (for isolation of RNA). Cell suspensions were stored at −80 °C, until subcellular fractions were prepared or the mRNA isolated.

### 4.5. Preparation of Subcellular Fractions

Microsomal and cytosolic fractions were obtained from the control and influenced Caco-2 cells suspended in a 0.1 M sodium phosphate buffer (pH 7.4). The cells were homogenized using sonication with Sonopuls (Bandelin, Germany). The subcellular fractions were isolated by differential centrifugation of the cell homogenate at 20,000× *g* for 60 min at 4 °C, with the resulting supernatant further centrifuged at 105,000× *g* (60 min, 4 °C). The supernatant and sediment from this step correspond to cytosol and microsomes, respectively. The microsomes were resuspended in a 0.1 M sodium phosphate buffer (pH 7.4) containing 20% glycerol (*v*/*v*). Subcellular fractions were stored at −80 °C.

### 4.6. Enzyme Assays

Cytosolic catechol-*O*-methyltransferase (COMT) activity was measured according to Aoyama et al. [34]. Cytosol (50 µL) was incubated with a 50 mM sodium phosphate buffer (pH 7.8) containing 2 mM MgCl_2_, 250 µM dithiothreitol, 200 µM SAM, and 100 µM norepinephrine in a volume of 125 µL for 1 h at 37 °C. The reaction was terminated by 12.5 µL of 1 M perchloric acid. The samples were left on ice for 5 min and centrifuged at 10,000× *g* for 5 min at 25 °C. The supernatant was used as a sample solution for HPLC analysis.

Cytosolic glutathione S-transferase (GST) activity was assayed according to Ye and Zhang [35]. Next, 194 µL of a 100 mM sodium phosphate buffer (pH 6.5) containing 1 mM GSH, 1 mM CDNB was added to 6 µL of the cytosolic fraction. Absorbance was measured at 340 nm by the Tecan Infinite M200.

A slightly modified version of the method of Frame et al. was used for the SULT activity assay [36]. The assay depends on the formation of 4-nitrophenol and 3′-phosphoadenosine-5′-phosphosulfate from 4-nitrophenolsulphate and PAP. The reaction mixture contained 5 mM MgCl_2_, 2 µM PAP, 5 mM 4-nitrophenylsulfate, and 100 µM 2-naphthol. After a 10-min incubation of 10 µL cytosol with 90 µL of reaction mixture, the reaction was stopped by 100 µL of 0.25 M Tris-HCl buffer (pH 8.7). Absorbance was measured at 405 nm by the Tecan Infinite M200.

Determination of the UDP-glucuronosyltransferase (UGT) activity is based on the method of Letelier et al. [37]. The microsomes (2 mg of protein/mL) were preincubated with a detergent for 20 min at 4 °C, at which time 90 µL of 2.5 mM *p*-nitrophenol, 1 mM UDP-glucuronic acid, 4 mM MgCl_2_, and 100 mM Tris HCl (pH 8.5) were added to 10 µL of the microsomes with detergent. The reaction was stopped after 20 min (37 °C) by 50 µL of 3% trichloroacetic acid. After proper mixing of the reaction mixture, 50 µL of the mixture was added to 50 µL of 1 M NaOH. Control samples were performed in the absence of UDP-glucuronic acid. Absorbance was monitored at 405 nm by the Tecan Infinite M200.

Catalytic activity of each enzyme was normalized to mg of protein in cytosolic/microsomal fractions. Protein concentrations in subcellular fractions were assayed using the bicinchoninic acid assay according to Sigma-Aldrich protocol.

### 4.7. RNA Isolation and Quantitative Real-Time PCR

Total RNA was isolated using TriReagent according to the manufacturer’s instructions. RNA concentration and purity was determined spectrophotometrically. First strand cDNA was synthesized from 1 µg of total RNA using ProtoScript II reverse transcriptase and random hexamers. qPCR analyses were performed in a QuantStudio 6 thermocycler (Applied Biosystems, Thermo Fisher Scientific, Waltham, MA, USA) using a qPCR Core kit for SYBR Green I following the manufacturer’s protocol. Primers were designed manually and are available upon request. Calculations were based on the “Delta-Delta Ct method” [38]. The data were expressed as the fold change of the treatment groups relative to the control.

### 4.8. Statistical Analysis

The data presented were obtained from three independent measurements. All calculations were done using Microsoft Excel and GraphPad Prism 6.04 (GraphPad Software, Inc., La Jolla, CA, USA). One-way ANOVA followed by a Dunnett’s test or Students *t*-test were used for the statistical evaluation of the differences between the treated groups and controls. *p*-Values < 0.05 were considered statistically significant.

## 5. Conclusions

Taken together, our observations demonstrated significantly elevated mRNA levels of main conjugation enzymes in differentiated Caco-2 cells versus undifferentiated counterparts, however, no differences in conjugation enzymes activities were observed. GTE/EGCG treatment did not affect the mRNA levels in either type of cells, although altered conjugation enzymes activity in differentiated cells was detected. These results indicate different modulatory effects of GTE and EGCG in expanding cancer cells (undifferentiated) and in enterocyte-like cells (differentiated). Given the data presented, based on the Caco-2 cell model, GTE mediated changes of GST and COMT activities in enterocyte-like cells were so mild that the risk of GTE interactions with concomitantly administered drugs seems to be low. However, the considerable beneficial effect of GTE via the pronounced induction of detoxifying enzymes cannot be expected as well.

## Figures and Tables

**Table 1:** Specific activities (nmol/min/mg of protein) and mRNA expression (normalized to GAPDH) of conjugation enzymes in proliferating (P) and differentiated (D) Caco-2 cells.

Enzyme	P	D
**GST**		
Activity	249 ± 13	268 ± 20
mRNA GSTA1/2	1.00 ± 0.04	*** 9.15 ± 0.45
mRNA GSTP1	1.00 ± 0.19	*** 2.60 ± 0.14
**SULT**		
Activity	20.7 ± 1.3	** 14.1 ± 1.6
mRNA SULT1A1/3	1.00 ± 0.03	*** 7.65 ± 0.33
**COMT**		
Activity	10.5 ± 0.9	12.9 ± 0.3
mRNA COMT	1.00 ± 0.09	*** 3.17 ± 0.14
**UGT**		
Activity	n.d.	n.d.
mRNA UGT1A	1.00 ± 0.09	*** 2.17 ± 0.08

Values represent means ± SD of three samples. Significance of differences of D cells from P cells was determined by a Students *t*-test (** *p* < 0.01, *** *p* < 0.001); n.d. = not detected. GAPDH: glyceraldehyde-3-phosphate dehydrogenase; GST: Glutathione S-transferases; SULT: Sulfotransferases; COMT: Catechol-*O*-methyltransferases; UGT: UDP-glucuronosyltransferases.

## Abbreviations

GTE: Green tea extract, Polyphenon 60
EGCG: (−)-Epigallocatechin gallate
GST: Glutathione S-transferases
UGT: UDP-glucuronosyltransferases
SULT: Sulfotransferases
COMT: Catechol-*O*-methyltransferases
P: Proliferating Caco-2 cells
D: Differentiated Caco-2 cells
NRU: Neutral red uptake
MTT: 3-(4,5-Dimethylthiazol-2-yl)-2,5-diphenyltetrazolium bromide
GAPDH: Glyceraldehyde 3-phosphate dehydrogenase
EMEM: Eagle’s minimum essential medium
SAM: S-(5′-Adenosyl)-l-methionine
GSH: l-Glutathione
CDNB: 1-Chloro-2,4-dinitrobenzene
PAP: 3′-Phosphoadenosine 5′-phosphate
DMSO: Dimethyl sulfoxide
PBS: Phosphate buffered saline
